# Unveiling the Hidden Burden: Psychiatric Comorbidities in Dialysis Patients

**DOI:** 10.1192/j.eurpsy.2025.1214

**Published:** 2025-08-26

**Authors:** M. Sant’Ovaia, J. M. de Castro, C. Martins, S. Martins, A. C. Ramos, T. M. Afonso

**Affiliations:** 1 Psychiatry, ULS Amadora Sintra, Lisboa, Portugal

## Abstract

**Introduction:**

Patients with End-Stage Renal Disease (ESRD) undergoing hemodialysis (HD) are particularly susceptible to psychological disorders, such as anxiety and depression, which can significantly affect their quality of life and clinical outcomes. However, in spite of its prevalence, psychiatric comorbidity in this population remains under-recognized, contributing to worse health outcomes and increased mortality.

**Objectives:**

The primary aim of this study is to explore the prevalence of psychiatric comorbidities, particularly depression and anxiety, among HD and PD patients. Insights into the different psychological effects of different dialysis treatments are offered, emphasizing the importance of integrated mental and physical healthcare in improving patient outcomes.

**Methods:**

A narrative review was carried out using PubMed and Google Scholar to identify relevant articles with the keywords “dialysis,” “psychiatry,” and “comorbidities.”

**Results:**

The review underscores the important burden of psychiatric disorders among dialysis patients. Studies suggest that depression may affect up to 50% of these individuals. Both HD and PD patients exhibit varying degrees of psychological distress, exacerbated by factors such as the invasiveness of dialysis, comorbid medical conditions (e.g., diabetes), and socioeconomic challenges. The evidence suggests that HD patients may experience an even more heightened psychiatric burden due to the more invasive nature of HD compared to PD, which imposes greater restrictions on daily activities. Psychiatric disorders in these patients are often underdiagnosed, leading to treatment non-compliance, increased hospital admissions, and elevated mortality rates. The findings stress the necessity of regular mental health screenings and the integration of psychiatric care into routine dialysis practice.

**Conclusions:**

Psychiatric comorbidities are prevalent among dialysis patients, with HD patients exhibiting a greater psychological burden. This review highlights the urgent need for routine mental health screening and intervention within the dialysis care framework. The integration of mental health support into dialysis treatment protocols could lead to improved patient outcomes, fewer hospitalizations, and better treatment adherence. Future research should focus on developing customized mental health interventions that address the specific challenges faced by dialysis patients, thereby enhancing their quality of life and clinical outcomes.

**Disclosure of Interest:**

None Declared

